# Evaluation of Normal Inferior Vena Cava Diameters in the Indian Adult Population by Computed Tomography

**DOI:** 10.7759/cureus.31845

**Published:** 2022-11-23

**Authors:** Ameen Ansari, Sarika Kohar, Avinash Dhok, Prashant Onkar, Kajal Mitra, Pooja Ladke

**Affiliations:** 1 Radiodiagnosis and Imaging, NKP Salve Institute of Medical Sciences and Research Centre, Nagpur, IND

**Keywords:** magnetic resonance imaging (mri), ultrasonography (us), vein, computed tomography (ct) scan, inferior vena cava (ivc)

## Abstract

Background

The imaging evaluation of inferior vena cava (IVC) diameters is essential for the estimation of vena caval pathologies and can also detect early hypovolemic shock. There are very few studies on normal IVC diameters on CT scan done in foreign countries, and none done in the Indian population.

Aims

The goal of this research is to assess the normal IVC diameter in the Indian adult population by performing a CT scan of the abdomen.

Material and methods

In this study, CT scans of 200 individuals (aged 19-83) without any circulatory and vascular disorders were analyzed retrospectively. The anteroposterior (AP) and transverse diameters of the IVC were measured at the level of the renal vein and at the level 2 cm proximal to insertion in the heart (usual area of measurement on ultrasonography).

Results

The study discovered normal adult mean AP and transverse dimensions of the IVC at the level of the renal vein as 16.3 ± 2.9 mm and 25.8 ± 3.5 mm, respectively, and 16.9 + 3.2 mm and 26.2 + 3.6 mm at the level 2 cm proximal to its insertion in the right atrium.

Conclusions

In this study, the normal morphometric dimensions of the IVC in the Indian adult population were established. The diameters of the IVC and the age of the participants in our study had no statistically significant correlation, however, the IVC changes its cross-sectional area and diameter due to changes in venous pressure and blood pressure and hence is a highly compliant vessel. The results of the study will be used as baseline data for the assessment of IVC disorders.

## Introduction

The inferior vena cava (IVC) is a large vein that supplies blood from the lower extremities and abdomen to the heart [1]. The use of imaging in the assessment and treatment of a range of disorders affecting the inferior vena cava is crucial [2]. Traditional venography, which was formerly the benchmark for detecting irregularities of the inferior vena cava, has been overtaken by non-invasive imaging methods for the detection of venous problems and is now prearranged for medicinal arbitration [3]. The use of high-resolution CT scans has aided in the diagnosis and treatment of cases having abdominal trauma dramatically [4]. Potentially a reliable indicator of hypovolemia in trauma patients is the size of the inferior vena cava on a CT scan [1]. Typically, dynamic multiphasic imaging is used to analyze the inferior vena cava during the portal venous phase, which lasts 60 to 70 seconds after the injection of 100 to 150 mL of nonionized contrast material at a rate of 3 to 5 mL/sec [2]. The renal and suprarenal inferior vena cava contrast during the portal venous phase is higher than it is in the infrarenal segment due to venous return from the kidneys [2]. A basic comprehension of physiologic and pharmacokinetic concepts, additionally as a comprehension of the outcome of injection parameters on arterial phase-both with regard to injection rate and injection time is required for the formulation of optimized contrast agent delivery protocols for multidetector computed tomography scanners [5]. There are some technological considerations. It's not always easy to foresee whether the inferior vena cava will play a key or secondary role in a disease process. In addition to screening people for abdominal symptoms that aren't specific, CT scans are frequently used to assess tumors that have the potential to spread to the inferior vena cava such as renal cell carcinoma, adrenocortical carcinoma, or hepatocellular carcinoma. Inferior vena cava malformations can occasionally be found by accident. The arterial phase, which is obtained when primary tumors are hypervascular or when a patient is getting ready for surgery, and the venous phase, which is obtained 60 to 70 seconds after injection, are two common CT sequences. It is more challenging to measure the infrarenal inferior vena cava and its confluent because the lower extremities' less opaque blood is present during the venous phase. However, because it exposes the patient to extra radiation, this additional imaging sequence cannot be usually justified [3]. Superior spatial resolution is combined with high-quality sagittal and coronal reformations and 3D volumetric projection due to the availability of high-speed CT scanners that enable the capture of near-isotropic data. Amazing photos of the healthy and unhealthy inferior vena cava are produced as a result [3]. The inferior vena cava may be a helpful predictor of volume levels in dialysis or kidney disease patients, according to a few case studies [6]. According to one study, abdominal trauma victims who showed a flat inferior vena cava on a CT scan were hypovolemic in six out of seven cases [4].

## Materials and methods

This is a hospital-based, retrospective, cross-sectional study performed in the department of radio-diagnosis in a tertiary care hospital in central India from January 2020 to December 2021. 200 patients who fit into the inclusion criteria entered the study. Inclusion criteria include patients of ages >18 years without any clinical or imaging signs of vascular or circulatory pathology undergoing a CT abdomen scan in the department of radiodiagnosis. The study includes out- and in-patients during the study period. Patients <18 years of age or having any vascular or circulatory pathology were excluded from the study.

CT scans were done using a Toshiba 16-slice machine (Toshiba Corporation, Tokyo, Japan). All data were collected and kept confidential, entered into a Microsoft Excel sheet (Microsoft Corporation, Redmond, WA), and utilized. A 0.5-2.5 mm detector collimator was one of the main methodology-based CT acquisition settings. The exposure parameters used in this study were 1.5 seconds of rotation, 120 kV, 100 mA, 10 mm increments, 3-10 mm slice thickness, and the same reconstruction index. If the thoracic and abdominal aortas were intact and accessible with soft tissue window settings, all scans of them were deemed appropriate.

The scan was typically initiated from the lower chest to the symphysis pubis, and contrast media (Omnipaque - 300 ml) was administered into the body through the venous system using the sure start technique, with a dose (70-100 ml) based on the patient's weight and age, a delay of 30-40 sec, and an injection rate of 2-3 ml/s using an automatic injector.

We measure the maximal anteroposterior and transverse diameters of the inferior vena cava at the level of the renal vein and 2 centimeters distal to the entry of the inferior vena cava into the right atrium. The caliber of the vessels will be ascertained using the length measurement equipment.

All parameters are presented with mean ± standard deviation (SD), maximum (Max), and minimum (Min) values. Statistical analysis was performed using the SPSS 20.0 software for Windows (IBM Corp., Armonk, NY). Levene’s test for equality of variance was used for statistical analysis. For the correlation of samples, Pearson's correlation and independent samples test was used.

The institutional ethics committee of NKP Salve Institute of Medical Sciences and Research Centre (Registration no. ECR/88/Inst/MH/2013/RR-19) issued approval IEC/06/2021 to conduct this study.

## Results

At the level of the renal vein, the mean AP diameter of the inferior vena cava was found to be 1.63 cm with an SD of 0.29 cm. The mean transverse diameter of the inferior vena cava was found to be 2.58 cm with an SD of 0.35 cm (Table 1).

**Table 1:** Descriptive statistics showing the mean inferior vena cava diameter at the level of the renal vein

Parameters	Min.	Max.	Mean	Std. Deviation	N
Age	19	83	51.3700	15.52347	200
AP Diameter	1	2.6	1.6270	.28860	200
TRANS Diameter	1.7	3.4	2.5800	.35159	200

In the present study, no significant association was observed between the age and the mean AP diameter of the inferior vena cava at the level of the renal vein (p=0.20). Similarly, no significant association was observed between the age and transverse diameter of the inferior vena cava at the level of the renal vein (p=0.31) (Table 2).

**Table 2:** Correlation between age and mean AP diameter, and age and mean TRANS diameter at the level of the renal vein AP: anteroposterior; TRANS: transverse

		Age	AP Diameter	TRANS Diameter
Age	Pearson Correlation	1	.091	.071
	Sig. (2-tailed)		.20	.315
	N	200	200	200
AP Diameter	Pearson Correlation	.091	1	.092
	Sig. (2-tailed)	.202		.197
	N	200	200	200
TRANS Diameter	Pearson Correlation	.071	.092	1
	Sig. (2-tailed)	.315	.19	
	N	200	200	200

**Figure 1:**
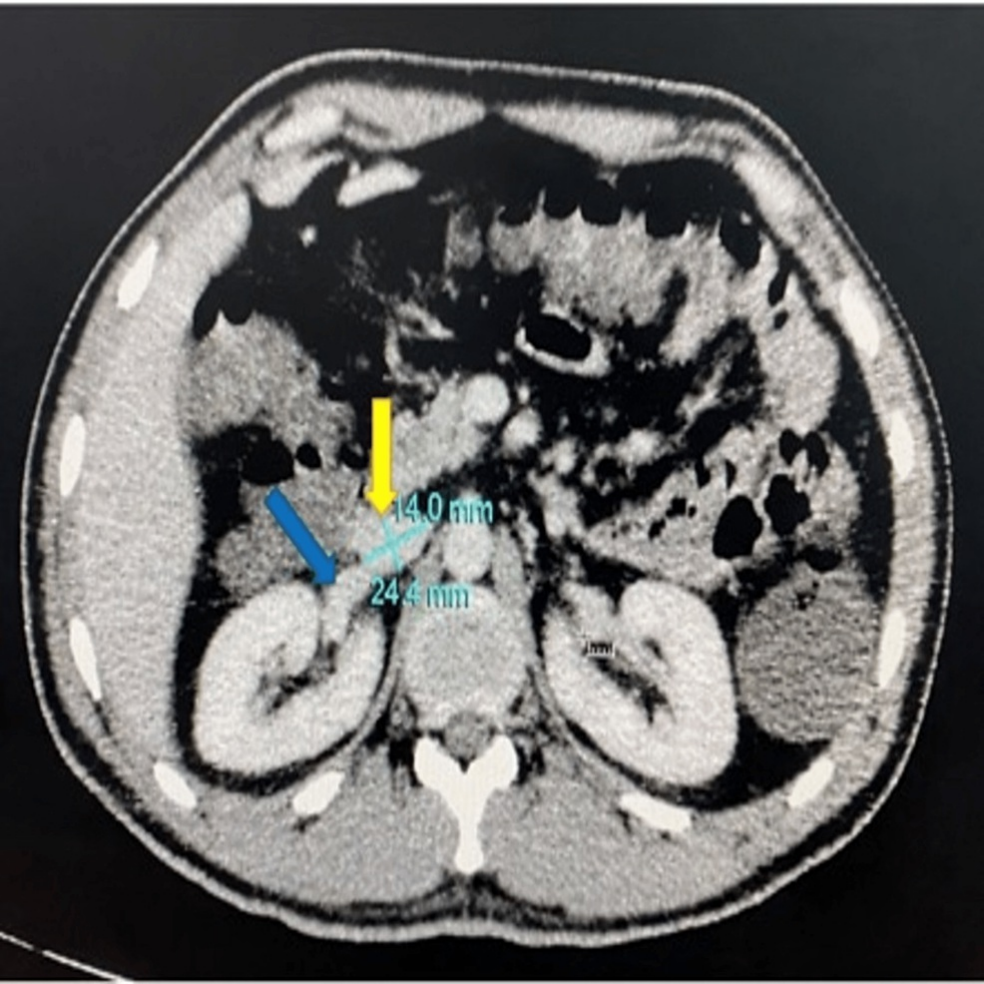
Axial CT scan image showing an inferior vena cava (yellow arrow) measurement taken at the level of the renal vein (blue arrow)

The axial CT scan image shows an inferior vena cava measurement taken at the level of the renal vein, where the AP diameter is 1.4 cm and the transverse (TRANS) diameter is 2.4 cm (Figure 1).

At the level 2 cm distal to the inferior vena cava insertion in the right atrium, the mean AP diameter of the inferior vena cava was found to be 1.69 cm with an SD of 0.32 cm. The mean transverse diameter of the inferior vena cava was found to be 2.62 cm with an SD of 0.36 cm (Table 3).

**Table 3:** Descriptive statistics showing the mean inferior vena cava diameter at the level 2 cm distal to its insertion in the right atrium

Parameters	Min.	Max.	Mean	Std. Deviation	N
Age	19	83	51.3700	15.52347	200
AP Diameter	1	2.8	1.6915	.32280	200
Trans Diameter	1.8	3.6	2.6515	.36367	200

In the present study, no significant association was observed between the age and the mean AP diameter of the inferior vena cava at 2 cm proximal to the insertion of the heart (p=0.35), Similarly, no significant association was observed between the age and TRANS diameter at 2 cm proximal to insertion heart (p=0.65). A negative correlation was observed between age and AP diameter at 2 cm proximal to insertion in the heart and age and TRANS diameter at 2 cm proximal to the insertion in the heart (Table 4).

**Table 4:** Correlation between age and mean AP diameter, and age and mean TRANS diameter at the level 2 cm proximal to its insertion in the heart AP: anteroposterior; TRANS: transverse

		Age	AP Diameter	Trans Diameter
Age	Pearson Correlation	1	-.065	-.032
	Sig. (2-tailed)		.357	.650
	N	200	200	200
AP Diameter	Pearson Correlation	-.065	1	.086
	Sig. (2-tailed)	.357		.226
	N	200	200	200
Trans Diameter	Pearson Correlation	-.032	.086	1
	Sig. (2-tailed)	.650	.226	
	N	200	200	200

**Figure 2:**
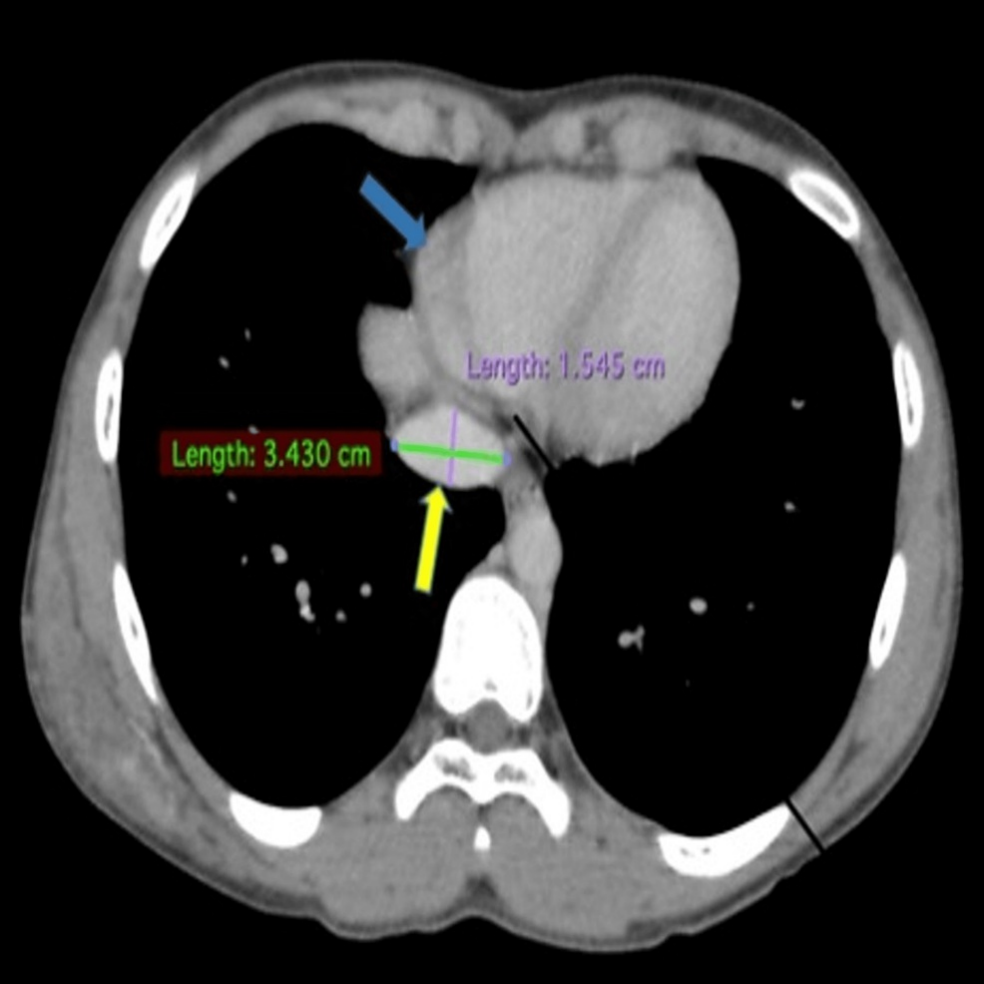
Axial CT scan image showing the inferior vena cava (yellow arrow) measurement taken at the level 2 cm distal to its insertion in the right atrium (blue arrow)

The axial CT scan image shows the inferior vena cava measurement taken at the level 2 cm distal to its insertion in the right atrium, where the AP diameter is 1.54 cm and the TRANS diameter is 3.45 cm (Figure 2).

At the renal vein, the mean observed AP diameter of the inferior vena cava was 1.63 cm, whereas at 2 cm proximal to the insertion of the heart, the mean observed was 1.69 cm (Table 5).

**Table 5:** Group statistics showing the mean difference between the AP diameter at the renal vein and at 2 cm of insertion in the heart AP: anteroposterior

	Groups	N	Mean	Std. Deviation	Std. Error Mean
AP Diameter	At renal vein	200	1.6270	.28860	.02041
	At 2 cm	200	1.6915	.32280	.02283

No significant association was observed between the AP diameter of the inferior vena cava at the renal vein and the AP diameter at 2 cm proximal to insertion in the heart (Table 6).

**Table 6:** Independent samples test showing significance in mean difference between the AP diameter of the inferior vena cava at the renal vein and the AP diameter at 2 cm proximal to insertion in the heart AP: anteroposterior

		Levene's Test for Equality of Variances		T-Test for Equality of Means						
		F	Sig.	T	df	Sig. (2-tailed)	Mean Difference	Std. Error Difference	95% Confidence Interval of the Difference	
									Lower	Upper
AP Diameter	Equal variances assumed	2.676	.103	-2.10	398	.036	-.06450	.03062	-.12469	-.00431
	Equal variances not assumed			-2.10	393.110	.036	-.06450	.03062	-.12469	-.00431

The mean observed TRANS diameter of the inferior vena cava at the renal vein was 2.58 cm, whereas at 2 cm proximal to insertion in the heart, the mean observed was 2.65 cm (Table 7).

**Table 7:** Group statistics showing the mean difference between the TRANS diameter at the renal vein and at 2 cm of insertion in the heart TRANS: transverse

	Groups	N	Mean	Std. Deviation	Std. Error Mean
TRANS Diameter	At renal vein	200	2.5800	.35159	.02486
	At 2 cm	200	2.6515	.36367	.02572

No significant association was observed between the TRANS diameter of the inferior vena cava at the renal vein and the TRANS Diameter at 2 cm proximal to the insertion in the heart (Table 8).

**Table 8:** Independent samples test showing significance in the mean difference between the TRANS diameter of the inferior vena cava at the renal vein and TRANS diameter at 2 cm proximal to insertion in the heart TRANS: transverse

		Levene's Test for Equality of Variances		t-test for Equality of Means						
		F	Sig.	t	df	Sig. (2-tailed)	Mean Difference	Std. Error Difference	95% Confidence Interval of the Difference	
									Lower	Upper
TRANS Diameter	Equal variances assumed	.01	.94	-1.99	398	.046	-.07150	.03577	-.14182	-.00118
	Equal variances not assumed			-1.99	397.547	.046	-.07150	.03577	-.14182	-.00118

## Discussion

The appearance of a collapsed inferior vena cava could be a key CT indication of hypovolemia caused by significant blood loss [4]. Women and older people were more likely to have a flattened inferior vena cava. The flat vena cava was most frequently detected in elderly women who did not show signs of hypotension or hypovolemia when it was restricted to one of the two levels evaluated in our analysis, notably at the level directly below the renal veins. Despite the fact that patients with physical trauma were more likely than patients without hypotension or hypovolemia to have a flattened inferior vena cava, 30% of patients showed overt indications of hypovolemia or hypotension, especially around the renal veins [7]. The presence of perivascular nodal masses and/or inferior vena cava invasion has prognostic significance and can aid surgical planning [8]. Tumor masses, breathing, and pathologic diseases that cause elevated right atrial and central venous pressures can all affect the size of the inferior vena cava [9]. Inferior vena cava measurement is useful for estimating dry weight in hemodialysis patients [10]. Reduced venous return is most likely to be responsible for the collapse of the inferior vena cava in hypovolemic patients [4]. Inferior vena cava diameters that are more than the upper limits of normal but do not meet aneurysm criteria should be referred to as dilated to prevent terminology ambiguity [1]. In reaction to blood volume and central venous pressure, the inferior vena cava alters its diameter and cross-sectional area [1]. The volume and width of the inferior vena cava can also be affected by respiratory, intrathoracic, and intra-abdominal pressures [10]. The subxiphoid transabdominal long-axis measurement is the most accurate ultrasonographic assessment of inferior vena cava diameter [11]. The IVC is a low-pressure, very flexible conduit, and changes in central venous pressure affect how it is configured [12]. The anabolic route is used to produce adenosine triphosphate (ATP) during times of tissue hypoxia, which results in the synthesis of lactate and hydrogen ions. As a result, lactate is frequently utilized as a sign of persistent shock [13]. One of the surgeon's top priorities when caring for injured patients is complete resuscitation from shock. Historically, the goal of resuscitation has been to restore the patient's blood pressure, heart rate, and urine output to normal [14]. Venacavography previously used to be the gold standard for identifying and assessing tumor thrombi [15]. Renal cell carcinoma (RCC) frequently invades the renal vein and may spread into the right atrium or inferior vena cava [16]. By using a transthoracic and subcostal view during echocardiography, the inferior vena cava can be easily visualized [17]. A great alternative for the initial evaluation is ultrasonography (US) with color Doppler flow imaging. It aids in the diagnosis and the representation of tumor thrombus and its extent using computed tomography (CT) and magnetic resonance imaging (MRI). Additionally, it aids in separating genuine thrombus from artificial filling flaws [2]. However, the US is operator-dependent and due to intestinal gas or obesity, the vision of the inferior vena cava (particularly the infrahepatic section) may be impeded. For staging and treatment planning, CT and MR imaging are required [2].

Our study had a few limitations. First, the sample size is small. There were only 200 patients, which may have impacted the precision of the estimate of the mean AP and transverse diameters of inferior vena cava, and the results cannot be generalized to the entire Indian population. Studies with larger sample sizes are needed in the future. Second, hydration status was not included in the analysis. This factor may impact the IVC dimensions, which could bias the results of this study.

## Conclusions

These normal inferior vena cava diameters can be used as a guide to assess whether the vein is distended or collapsed in a variety of illnesses and pathologies. The diameter of the inferior vena cava can be measured 2 cm distal to the entry of the inferior vena cava into the right atrium (the normal location for ultrasonographic measurement) as well as at the level of the renal vein with no discernible change. The findings of this study will help radiologists detect and properly define inferior vena cava anomalies, allowing them to make better clinical decisions and improve patient care.

## References

[1] Elbagir S, Yousef M, Abdelaziz Hassan I, Bushara L, Abdoh H, Salih M (2018). Evaluation of the normal inferior vena cava diameters in Sudanese’s by multidetector computed tomography. https://www.researchgate.net/publication/327103848_Evaluation_of_the_Normal_Inferior_Vena_Cava_Diameters_in_Sudanese's_by_Multidetector_Computed_Tomography.

[2] Kandpal H, Sharma R, Gamangatti S, Srivastava DN, Vashisht S (2008). Imaging the inferior vena cava: a road less traveled. Radiographics.

[3] Sheth S, Fishman EK (2007). Imaging of the inferior vena cava with MDCT. AJR Am J Roentgenol.

[4] Jeffrey RB Jr, Federle MP (1988). The collapsed inferior vena cava: CT evidence of hypovolemia. AJR Am J Roentgenol.

[5] Fleischmann D (2003). High-concentration contrast media in MDCT angiography: principles and rationale. Eur Radiol.

[6] Naruse M, Sakaguchi S, Nakayama Y, Nonoguchi H, Tomita K (2007). A novel method for dry weight assessment in hemodialysis patients: utilization of inferior vena cava flat ratio to correct for individual variations in vessel diameter. Ther Apher Dial.

[7] Eisenstat RS, Whitford AC, Lane MJ, Katz DS (2002). The "flat cava" sign revisited: what is its significance in patients without trauma?. AJR Am J Roentgenol.

[8] Gosink BB (1978). The inferior vena cava: mass effects. AJR Am J Roentgenol.

[9] Mintz GS, Kotler MN, Parry WR, Iskandrian AS, Kane SA (1981). Real-time inferior vena caval ultrasonography: normal and abnormal findings and its use in assessing right-heart function. Circulation.

[10] Nakao S, Come PC, McKay RG, Ransil BJ (1987). Effects of positional changes on inferior vena caval size and dynamics and correlations with right-sided cardiac pressure. Am J Cardiol.

[11] Finnerty NM, Panchal AR, Boulger C (2017). Inferior vena cava measurement with ultrasound: what is the best view and best mode?. West J Emerg Med.

[12] Natori H, Tamaki S, Kira S (1979). Ultrasonographic evaluation of ventilatory effect on inferior vena caval configuration. Am Rev Respir Dis.

[13] Huckabee WE (1961). Abnormal resting blood lactate. I. The significance of hyperlactatemia in hospitalized patients. Am J Med.

[14] Porter JM, Ivatury RR (1998). In search of the optimal end points of resuscitation in trauma patients: a review. J Trauma.

[15] Hallscheidt PJ, Fink C, Haferkamp A (2005). Preoperative staging of renal cell carcinoma with inferior vena cava thrombus using multidetector CT and MRI: prospective study with histopathological correlation. J Comput Assist Tomogr.

[16] Lawrentschuk N, Gani J, Riordan R, Esler S, Bolton DM (2005). Multidetector computed tomography vs magnetic resonance imaging for defining the upper limit of tumour thrombus in renal cell carcinoma: a study and review. BJU Int.

[17] Patil S, Jadhav S, Shetty N, Kharge J, Puttegowda B, Ramalingam R, Cholenahally MN (2016). Assessment of inferior vena cava diameter by echocardiography in normal Indian population: a prospective observational study. Indian Heart J.

